# Malignant fibrous neoplasms of long bones: analysis of the surveillance, epidemiology, and end results database from 1973 to 2015

**DOI:** 10.1186/s12891-019-2971-8

**Published:** 2020-01-22

**Authors:** Yiting Huang, Jianqiao Hong, Jiahong Meng, Haobo Wu, Mingmin Shi, Shigui Yan, Wei Wang

**Affiliations:** 1 Division of Reproductive Medicine & Infertility, The Second Affiliated Hospital, Zhejiang University School of Medicine, No.88 Jiefang Road, Hangzhou, 310009 People’s Republic of China; 2 Department of Orthopaedic Surgery, The Second Affiliated Hospital, Zhejiang University School of Medicine, No.88 Jiefang Road, Hangzhou, 310009 People’s Republic of China

**Keywords:** Malignant fibrous neoplasms (MFN), Fibrosarcoma, Fibromyxosarcoma, Periosteal fibrosarcoma, Malignant fibrous histiocytoma, Long bones, Prognosis

## Abstract

**Background:**

Malignant fibrous neoplasms (MFN) of long bones are rare lesions. Moreover, the prognostic determinants of MFN of long bones have not been reported. This study aimed to present epidemiological data and analyse the prognostic factors for survival in patients with MFN.

**Materials and methods:**

The Surveillance, Epidemiology, and End Results (SEER) programme database was used to screen patients with malignant fibrous neoplasms (MFN) of long bones from 1973 to 2015, with attention to fibrosarcoma, fibromyxosarcoma, periosteal fibrosarcoma and malignant fibrous histiocytoma. The prognostic values of overall survival (OS) and cancer-specific survival (CSS) were assessed using the Cox proportional hazards regression model with univariate and multivariate analyses. The Kaplan–Meier method was used to obtain OS and CSS curves.

**Results:**

A total of 237 cases were selected from the SEER database. Malignant fibrous histiocytoma was the most common form of lesion in long bones. Multivariate analysis revealed that independent predictors of OS included age, stage, tumour size and surgery. Age, stage, tumour size and surgery were also independent predictors of CSS. Additionally, the most significant prognostic factor was whether metastasis had occurred at the time of initial diagnosis.

**Conclusion:**

Among patients with MFN of long bones, age (> 60 years), tumour size (> 10 cm), distant stage, and non-surgical treatment are factors for poor survival.

## Background

Malignant fibrous neoplasms (MFN) are very rare malignant tumours of long bones. These tumours include fibrosarcoma, fibromyxosarcoma, periosteal fibrosarcoma and malignant fibrous histiocytoma (MFH) according to classification from the SEER database. Malignant fibrous neoplasms most commonly arise from the extremities [1], occasionally from the nasal cavity and paranasal sinuses [2], and rarely from long bone.

MFH was first described by O’Brien in 1963 [3] and by Kauffman and Stout (Histiocytic tumors (fibrous xanthoma and histiocytoma) in children in 1961 [4]. It was the most common soft tissue sarcoma [5]. Fibrosarcoma of long bone (LBFS) is another neoplasm in this region that exhibits nonspecific symptoms such as pain. The final diagnosis of this tumour is based on histopathology and immunohistochemistry [6].

Malignant tumours of the long bones are challenging with regard to improving patient survival [7]. Few studies have reported the complications associated with surgical resection. Therefore, it remains unresolved whether a uniform treatment strategy could be beneficial to all patients.

Here, we present an epidemiologic analysis of MFN of long bone using the U.S. National Cancer Institute’s SEER database from 1973 to 2015. No previous study has specifically performed an in-depth analysis of cases with MFN of long bones using the database. We analysed 237 cases of MFN of long bones from 18 SEER registries, representing the largest cohort of patients with MFN of long bones studied to date. Our analysis includes demographic and clinicopathologic features of this rare neoplasm, along with its survival outcomes.

## Materials and methods

Frequency and survival data were obtained from the SEER 18 dataset from 1973 to 2015. SEER had considerably fewer participating registries in the early years, and the number gradually increased relatively recently to reach 18. The cases included in this dataset were diagnosed between 1973 and 2015. Fibrosarcoma cases were screened using the morphological codes for fibrosarcoma (8810/3), fibromyxosarcoma (8811/3), periosteal fibrosarcoma (8812/3), and malignant fibrous histiocytoma (8830/3). Based on the topographical codes, cases with MFN were restricted to the long bone, namely, the long bones of the upper limb, scapula, and associated joints (C40.0), as well as the long bone of the lower limb and associated joints (C40.2).

Frequency data were stratified and analysed by age, gender, race, grade, tumour size, SEER extent of disease, and treatment strategy. SEER extent of disease is categorized into localized, regional, and distant disease. Localized tumour, regional tumour and distant disease are defined as previously reported [8–11]. Five-year survival rates were calculated using Kaplan-Meier analysis, calculating overall survival (OS) and calculating disease-specific survival (CSS) rates [12]. SEER*Stat 8.1.5 software (National Cancer Institute, Bethesda, MD) and Microsoft Excel 2013 (Microsoft Corp., Redmond, WA) were used to process the data. Statistical Product and Service Solutions (SPSS) 24th edition was used to yield Kaplan-Meier curves and CSS rates. Probability values (*p* values) *<* 0.05 were considered statistically significant for all tests.

## Results

The demographic characteristics of the 237 patients with MFN of the long bones identified in the SEER database are displayed in Table 1. The specific histopathological diagnosis was fibrosarcoma (21.5%), fibromyxosarcoma (1.7%), periosteal fibrosarcoma (5.1%), and malignant fibrous histiocytoma (71.7%).

**Table 1:** Clinical characteristics of patients with MFN of long bones

Variables		Number (%)				
		Fibrosarcoma (NOS) *N* = 51	Fibromyxosarcoma *N* = 4	Periosteal fibrosarcoma *N* = 12	Malignant fibrous histiocytoma *N* = 170	Total
		57	61	48	56	56
Age	≤16	1 (2.0%)	0 (0%)	2 (16.7%)	9 (5.3%)	12 (5.1%)
	17–39	11 (21.6%)	1 (25.0%)	2 (16.7%)	28 (16.5%)	42 (17.7%)
	40–60	16 (31.4%)	0 (0.0%)	3 (25.0%)	54 (31.8%)	73 (30.8%)
	>60	23 (45.1%)	3 (75.0%)	5 (41.7%)	79 (46.5%)	110 (46.4%)
Race recode	Other	4 (7.8%)	0 (0.0%)	3 (25.0%)	9 (5.3%)	16 (6.8%)
	White	39 (76.5%)	4 (100.0%)	9 (75.0%)	143 (84.1%)	195 (82.3%)
	Black	8 (15.7%)	0 (0.0%)	0 (0.0%)	18 (10.6%)	26 (11.0%)
Sex	Female	19 (37.3%)	2 (50.0%)	6 (50.0%)	82 (48.2%)	109 (46.0%)
	Male	32 (62.7%)	2 (50.0%)	6 (50.0%)	88 (51.8%)	128 (54.0%)
Decade of diagnosis	1970s	8 (15.7%)	0 (0.0%)	7 (58.3%)	7 (4.1%)	22 (9.3%)
	1980s	7 (13.7%)	0 (0.0%)	3 (25.0%)	30 (17.6%)	40 (16.9%)
	1990s	6 (11.8%)	0 (0.0%)	0 (0.0%)	38 (22.4%)	44 (18.6%)
	2000s	17 (33.3%)	3 (75.0%)	1 (8.3%)	73 (42.9%)	94 (39.7%)
	2010s	13 (25.5%)	1 (25.0%)	1 (8.3%)	22 (12.9%)	37 (15.6%)
Tumor grade	Unknown	7 (13.7%)	1 (25.0%)	6 (50.0%)	63 (37.1%)	77 (32.5%)
	Low	18 (35.3%)	1 (25.0%)	3 (25.0%)	7 (4.1%)	29 (12.2%)
	High	26 (51.0%)	2 (50.0%)	3 (25.0%)	100 (58.8%)	131 (55.3%)
Tumor Size	<5 cm	27 (52.9%)	0 (0.0%)	9 (75.0%)	83 (48.8%)	119 (50.2%)
	5 cm-10	8 (15.7%)	0 (0.0%)	0 (0.0%)	18 (10.6%)	26 (11.0%)
	>10	13 (25.5%)	4 (100.0%)	2 (16.7%)	42 (24.7%)	61 (25.7%)
	Unknown	3 (5.9%)	0 (0.0%)	1 (8.3%)	27 (15.9%)	31 (13.1%)
Stage	Unknown	4 (7.8%)	0 (0.0%)	1 (8.3%)	11 (6.5%)	16 (6.8%)
	Local	23 (45.1%)	2 (50.0%)	4 (33.3%)	62 (36.5%)	91 (38.4%)
	Region	16 (31.4%)	1 (25.0%)	5 (41.7%)	62 (36.5%)	84 (35.4%)
	distant	8 (15.7%)	1 (25.0%)	2 (16.7%)	35 (20.6%)	46 (19.4%)
Surgery	No	13 (19.4%)	0 (0.0%)	5 (41.7%)	38 (22.4%)	56 (23.6%)
	Yes	38 (74.5%)	4 (100.0%)	7 (58.3%)	131 (77.1%)	180 (75.9%)
	Unknown	0 (0.0%)	0 (0.0%)	0 (0.0%)	1 (0.6%)	1 (0.4%)
Radiotherapy	No/Unknown	36 (70.6%)	4 (100.0%)	8 (66.7%)	133 (78.2%)	181 (76.4%)
	Yes	15 (29.4%)	0 (0.0%)	4 (33.3%)	37 (21.8%)	56 (23.6%)
Chemotherapy	No/Unknown	36 (70.6%)	3 (75.0%)	8 (66.7%)	87 (51.2%)	134 (56.5%)
	Yes	15 (29.4%)	1 (25.0%)	4 (33.3%)	83 (48.8%)	103 (43.5%)
Dead	No	15 (29.4%)	3 (75.0%)	3 (25.0%)	56 (32.9%)	77 (32.5%)
	Yes	36 (70.6%)	1 (25.0%)	9 (75.0%)	114 (67.1%)	160 (67.5%)
Tumor sequence	First	39 (76.5%)	4 (100.0%)	12 (100.0%)	153 (90.0%)	208 (87.8%)
	≥Second	12 (23.5%)	0 (0.0%)	0 (0.0%)	17 (10.0%)	29 (12.2%)
5-year OS rate		31.40%	50.00%	58.30%	40.60%	39.70%
5-year CSS rate		62.70%	75.00%	66.70%	58.80%	60.30%
10-year OS rate		11.80%	50.00%	50.00%	23.50%	22.80%
10-year CSS rate		58.80%	75.00%	58.30%	55.30%	56.50%

*Abbreviations*: *CSS* Cancer-specific survival, *OS* Overall survival, *MFN* Malignant fibrous neoplasms

Histologically, 55.3% of cases were high grade, 12.2% were low grade, and 32.5% were an unknown tumour grade. More than half (50.2%) of the tumours were at less than 5 cm in size. The extent of disease showed that the majority presented with locally invasive disease (38.4%). Forty-six patients (19.4%) had developed metastasis at presentation. Most patients were diagnosed first with malignant primary tumours (87.8%). After diagnosis, 75.9% of patients underwent surgical treatment and 56.5% of the patients underwent chemotherapy. A total of 160 patients (67.5%) died. The OS rates of the entire cohort at 5 and 10 years were 39.7 and 22.8%, respectively. The CSS rates at 5 and 10 years were 60.3 and 56.5%, respectively.

The 5- and 10-year OS rates for patients with fibrosarcoma of long bones were 31.4 and 11.8%, respectively, and the respective 5- and 10-year CSS rates were 62.7 and 58.8%. For patients with malignant fibrous histiocytoma of long bones, the OS rates were 40.6 and 23.5% and the CSS rates were 58.8 and 55.3%, respectively (Table 1).

Univariate and multivariate analysis of parameters that influence overall survival (OS) and cancer-specific survival (CSS) are illustrated in Tables 2 and 3.

**Table 2:** Univariate and multivariate analyses for OS for patients identified in the SEER Program database from 1973 to 2015

Variables	Univariate analysis			Multivariate analysis	
		*p*-value	Hazard ratio (95% CI)	*p*-value	Hazard ratio (95% CI)
Age	≤16	/	1	/	1
	17–39	0.124	2.562	0.098	2.765 (0.827–9.243)
	40–60	0.037*	3.47 (1.075–11.202)	0.027*	3.81 (1.163–12.486)
	>60	0.001*	7.381 (2.323–23.45)	0.004*	5.688 (1.722–18.782)
Sex	Male	/	1		
	Female	0.276	0.840 (0.613–1.150)		
Decade of diagnosis	1970s	/	1		
	1980s	0.938	0.977 (0.552–1.731)		
	1990s	0.204	1.444 (0.819–2.545)		
	2000s	0.382	0.784 (0.454–1.353)		
	2010s	0.999	1 (0.512–1.954)		
Race recode	Other	/	1		
	White	0.435	1.293 (0.679–2.462)		
	Black	0.505	1.301 (0.6–2.822)		
Tumor Grade	Low	/	1		
	High	0.065	1.691 (0.968–2.954)		
	Unknown	0.02*	1.97 (1.115–3.48)		
Tumor type	Fibrosarcoma (NOS)	/	1		
	Fibromyxosarcoma	0.188	0.263 (0.036–1.923)		
	Periosteal fibrosarcoma	0.249	0.649 (0.311–1.354)		
	Malignant fibrous histiocytoma	0.609	0.906 (0.621–1.322)		
Tumor Size	<5 cm	/	1	/	1
	5 cm-10	0.109	1.889 (0.868–4.114)	0.034*	2.335 (1.065–5.124)
	>10	< 0.001*	4.714 (2.111–10.527)	0.001*	4.051 (1.769–9.275)
	Unknown	< 0.001*	3.301 (1.602–6.801)	0.027*	2.324 (1.1–4.91)
Tumor sequence	First	/	1	/	1
	≥Second	0.031*	1.618 (1.045–2.505)	0.142	1.425 (0.889–2.287)
Stage	Local	/	1	/	1
	region	0.128	1.355 (0.916–2.004)	0.091	1.421 (0.945–2.135)
	distant	< 0.001*	5.383 (3.517–8.237)	< 0.001*	3.917 (2.427–6.321)
	unknown	< 0.001*	2.68 (1.468–4.892)	0.515	1.239 (0.65–2.359)
Surgery	No	/	1	/	1
	Yes	< 0.001*	0.267 (0.19–0.376)	< 0.001*	0.397 (0.262–0.6)
	unknown	0.408	0.433 (0.06–3.144)	0.528	0.515 (0.066–4.05)
Radiotherapy	no/unknown	/	1	/	1
	Yes	0.003*	1.684 (1.196–2.371)	0.831	0.959 (0.655–1.404)
Chemotherapy	no/unknown	/	1	/	1
	Yes	0.023*	0.688 (0.499–0.949)	0.282	0.816 (0.563–1.182)

*Abbreviations*: *OS* Overall survival, *SEER* Surveillance, epidemiology, and end results

*p < 0.05

**Table 3:** Univariate and multivariate analyses for CSS for patients identified in the SEER Program database from 1973 to 2015

Variables	Univariate analysis			Multivariate analysis	
		*p*-value	Hazard ratio (95% CI)	*p*-value	Hazard ratio (95% CI)
Age	≤16	/	1	/	1
	17–39	0.192	2.243 (0.666–7.554)	0.1	2.803 (0.822–9.556)
	40–60	0.206	2.153 (0.655–7.074)	0.183	2.271 (0.679–7.601)
	>60	0.032*	3.569 (1.112–11.454)	0.03*	3.863 (1.14–13.091)
Sex	Male	/	1		
	Female	0.427	0.854 (0.578–1.261)		
Decade of diagnosis	1970s	/	1		
	1980s	0.82	1.088 (0.526–2.249)		
	1990s	0.447	1.323 (0.643–2.72)		
	2000s	0.358	0.726 (0.367–1.437)		
	2010s	0.514	0.755 (0.324–1.759)		
Race recode	Other	/	1		
	White	0.853	1.076 (0.497–2.329)		
	Black	0.28	1.633 (0.671–3.974)		
Tumor Grade	High	/	1		
	Low	0.101	1.859 (0.885–3.905)		
	Unknown	0.06	2.079 (0.969–4.464)		
Tumor type	Fibrosarcoma (NOS)	/	1		
	Fibromyxosarcoma	0.436	0.45 (0.061–3.351)		
	Periosteal fibrosarcoma	0.571	0.754 (0.284–2.004)		
	Malignant fibrous histiocytoma	0.864	1.043 (0.643–1.693)		
Tumor Size	<5 cm	/	1	/	1
	5 cm-10	0.163	1.446 (0.861–2.426)	0.205	1.908 (0.703–5.176)
	>10	< 0.001*	7.595 (4.553–12.671)	0.033*	3.075 (1.092–8.659)
	Unknown	0.08	2.108 (0.914–4.863)	0.082	2.314 (0.899–5.951)
Tumor sequence	First	/	1	/	1
	≥Second	0.023*	0.042 (0.003–0.641)	0.943	0
Stage	local	/	1	/	1
	region	0.128	1.355 (0.916–2.004)	0.145	1.481 (0.873–2.514)
	distant	< 0.001*	5.383 (3.517–8.237)	< 0.001*	4.401 (2.47–7.841)
	unknown	< 0.001*	2.68 (1.468–4.892)	0.942	0.967 (0.396–2.363)
Surgery	No	/	1	/	1
	Yes	< 0.001*	0.226 (0.15–0.34)	< 0.001*	0.318 (0.191–0.527)
	unknown	0.963	0	0.989	0
Radiotherapy	no/unknown	/	1	/	1
	Yes	0.02*	1.652 (1.084–2.517)	0.589	0.878 (0.546–1.409)
Chemotherapy	no/unkown	/	1	/	1
	yes	0.481	0.87 (0.59–1.282)	0.871	0.963 (0.609–1.523)

*Abbreviations*: *CSS* Cancer-specific survival, *SEER* Surveillance, epidemiology, and end results

*p < 0.05

**Fig. 1:**
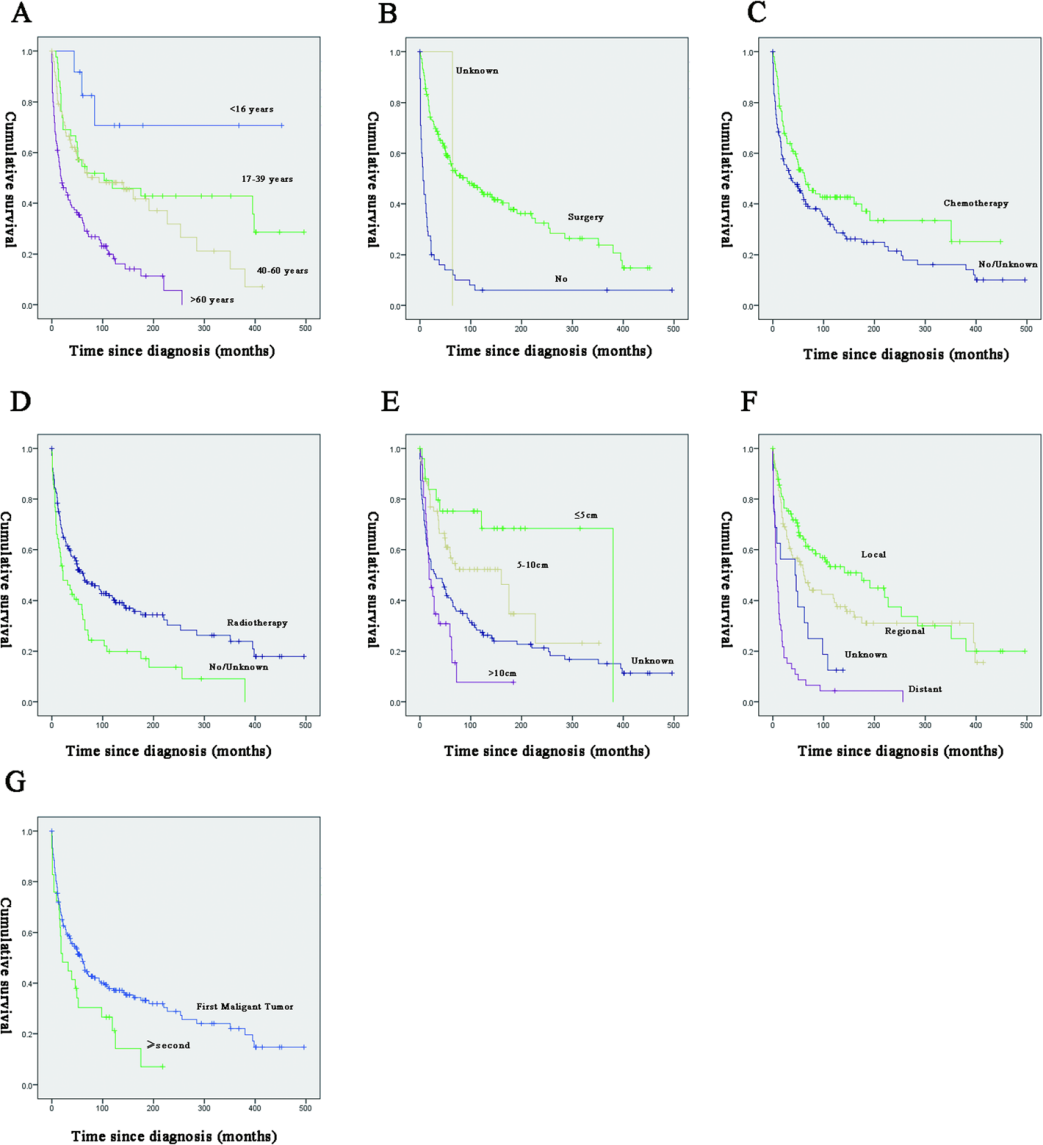
Kaplan–Meier method estimated OS in patients with MFN of the long bones stratified by **a** age at diagnosis (years), **b** surgery, **c** chemotherapy or not, **d** radiation treatment, **e** tumour size, **f** stage, **g** tumor sequence. Abbreviations: OS, overall survival; MFN, malignant fibrous neoplasms

**Fig. 2:**
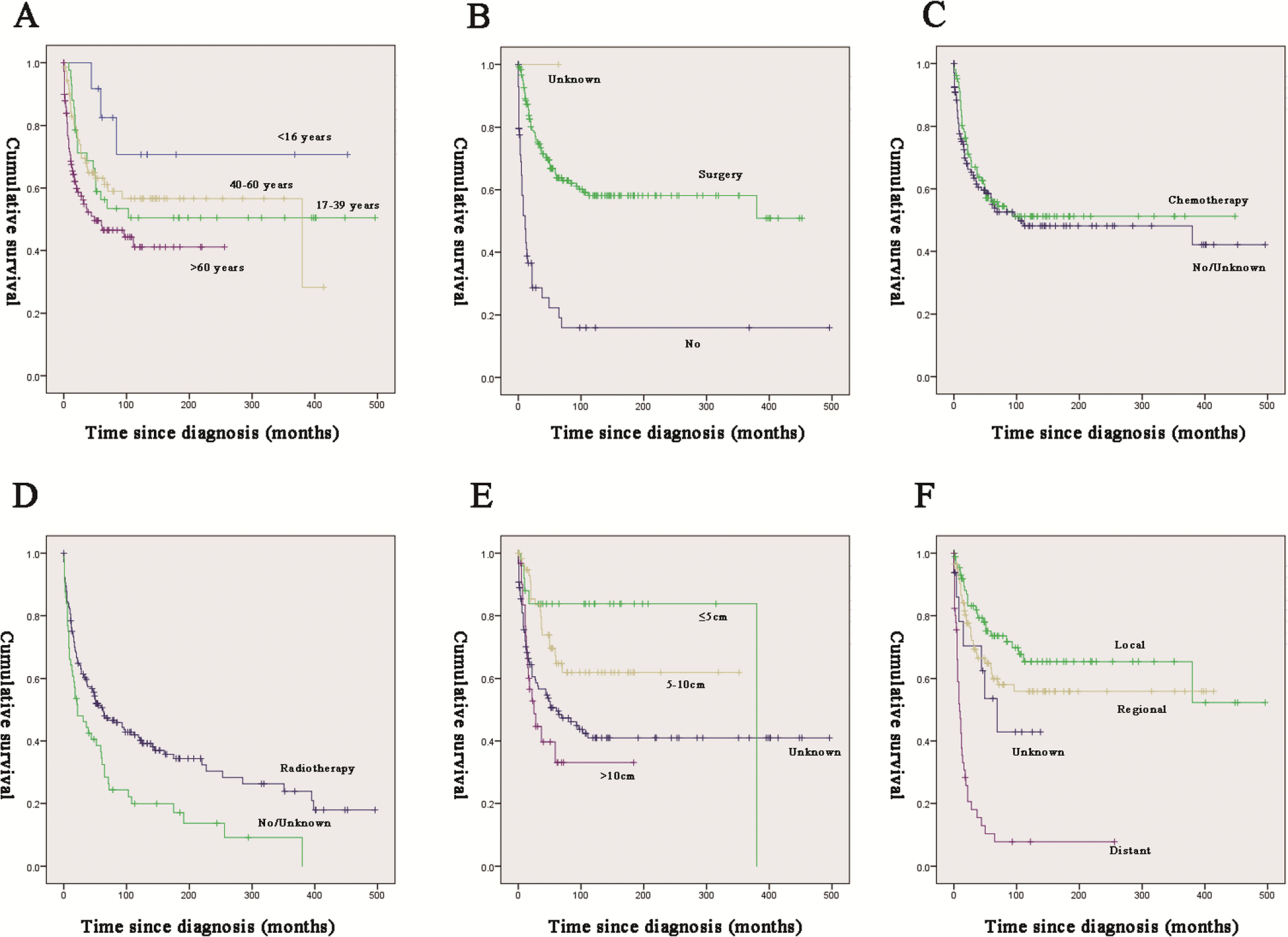
Kaplan–Meier method estimated CSS in patients with MFN of the long bones stratified by **a** age at diagnosis (years), **b** surgery, **c** chemotherapy or not, **d** radiation treatment, **e** tumour size, **f** stage. Abbreviations: CS, cancer-specific survival; MFN, malignant fibrous neoplasms

For both OS and CSS, race, gender, decade of diagnosis and tumour type showed no significant effect on survival (*p* > 0.05; Tables 2 and 3). Univariate survival analysis revealed that older age (age > 60 years) was significantly associated with a worse OS (HR = 7.381, *p* = 0.001; Table 2; Fig. 1a) and CSS (HR = 3.569, *p* = 0.032; Table 3; Fig. 2a). Tumour size (> 10 cm) was significantly associated with a worse OS (HR = 4.714, *p* < 0.001; Table 2; Fig. 1e) and CSS (HR = 7.595, *p* < 0.001; Table 3; Fig. 2e). Tumour sequence (second) was significantly associated with a worse OS (HR = 1.618, *p* = 0.031; Table 2) but a higher CSS (HR = 0.042, *p* = 0.023; Table 3). The stage (distant) was significantly associated with a worse OS (HR = 5.383, *p* < 0.001; Table 2; Fig. 1f) and CSS (HR = 5.383, *p* < 0.001; Table 3; Fig. 2f). Radiotherapy was significantly associated with a worse OS (HR = 1.684, *p* = 0.003; Table 2; Fig. 1d) and CSS (HR = 1.652, *p* = 0.02; Table 3; Fig. 2d). Surgery was significantly associated with a better OS (HR = 0.267, *p* < 0.001; Table 2; Fig. 1b) and CSS ((HR = 0.267, *p* = 0.226; Table 3; Fig. 2b). Chemotherapy was significantly associated with a better OS (HR = 0.688, *p* = 0.023; Table 2; Fig. 1c) but not with CSS (HR = 0.226, *p* < 0.001; Table 3; Fig. 2c).

Upon multivariate analysis of all patients (Table 2), age (> 60 years vs < 16 years, HR = 5.688; *p* = 0.004; 40–60 years vs < 16 years, HR = 3.81; *p* = 0.027), stage (distant vs. localized, HR = 3.917, *p* < 0.001), tumour size (> 10 cm vs < 5 cm, HR = 4.051; *p* = 0.001; 5–10 cm vs < 5 cm, HR = 2.335; *p* = 0.034), and surgery (HR = 0.397; *p* < 0.001) were independent predictors of OS.

The results of the multivariate analysis of the parameters that influence CSS are presented in Table 3. Age (> 60 years vs < 16 years, HR = 3.863; 95% CI, 1.14–13.091; *p* = 0.03), stage (distant vs. localized, HR = 4.401, 95% CI, 2.47–7.841, *p* < 0.001), tumour size (> 10 cm vs < 5 cm, HR = 3.075; 95% CI, 1.092–8.659; *p* = 0.033; 5–10 cm vs < 5 cm, HR = 2.335; 95% CI, 1.065–5.124; *p* = 0.034), and surgery (HR = 0.318; 95% CI, 0.191–0.527; *p* < 0.001) were independent predictors of CSS.

## Discussion

MFN are rare malignant tumours. Attention was paid to fibrosarcoma, fibromyxosarcoma, periosteal fibrosarcoma and malignant fibrous histiocytoma.

These tumours can infiltrate adjacent tissues and metastasize distally [13–19]. However, the outcome and prognosis of patients with MFN of long bones have not been reported because of its rarity. To our knowledge, the current study is the first to report the prognostic factors that affect the survival of patients with MFN of long bones using multivariate regression analysis.

In the current study, we identified 237 cases of malignant fibrous neoplasms of long bone based on the SEER database from 1973 to 2015. The OS and CSS rates at 5 years were 39.7 and 60.3%, respectively, while the previous study results showed that the OS and CSS of sinonasal fibrosarcoma were 71.7 and 77.8%, respectively [13].

Although malignant fibrous histiocytoma (MFH) ceased to be recognised as an entity in the 2013 edition of the WHO classification of bone and soft tissue tumours [20], undifferentiated high-grade pleomorphic sarcoma is listed as a new synonym for MFH of bone, still with the same code 8830/3, in the recently published ICD-O-3.2. However, the registrations of undifferentiated high-grade pleomorphic sarcoma of bone still decreased in the seer database. This might explain the recent decrease in registrations of MFH. Therefore, this study was of more than purely historical interest.

### Survival by age

We observed different survival rates by age at diagnosis in many studies [14]. In contrast, we found a higher risk of poor OS in the elder group. In the multivariate analysis of OS for patients aged 17–39 years the HR was 2.765 (*P* = 0.098), for those aged 40–60 years the HR was 3.81 (*P* = 0.027), and for those aged > 60 years the HR was 5.688 (*P* = 0.004). We also found a higher risk of poor CSS in the elder group. In the multivariate analysis of CSS for those aged > 60 years, the HR was 3.863 (*P* = 0.03*).

### Survival by gender, race and decade of diagnosis

Our results were consistent with previous results where no differences in survival by gender, or race but not decades were founded [21]. The differences of decades might be caused by the primary site of long bone or small bone.

### Survival by radiotherapy and chemotherapy

Radiotherapy was used as an adjunct to surgical management in patients with positive margins [22]. In the univariate analysis of OS, radiotherapy (RT) was associated with poor CSS (HR was 1.652(*P* = 0.02)) and OS (HR was 1.684(*P* = 0.003)). The results were consistent with a recent study [15]. This may be caused by the selection bias of patients with radiotherapy. Chemotherapy was also an important adjuvant therapy. In the multivariate analysis of OS and CSS, we did not find that chemotherapy was an independent prognostic factor for OS and CSS. However, a previous study demonstrated that clinical factors were associated with radiotherapy only in nonmetastatic malignant fibrous histiocytoma (MFH) of soft tissues [1].

### Survival for tumour type and tumour sequence

Tumour type was not an independent prognostic indicator for OS or CSS in multivariate analysis. In addition, tumour sequence was an independent prognostic indicator of OS and CSS, suggesting that patients with second primary bone MFN in their long bones may have a worse prognosis than those with a first primary tumour. The patients’ number of more than 2 sequence was as low as 29, among which 12 in fibrosarcoma, 17 in malignant fibrous histiocytoma. This leads to the difficulty in analyzing in multivariate analysis. Therefore, the results of tumour sequence should be taken into consideration carefully.

### Survival by tumour size

For smaller tumours with no evidence of metastasis, surgical extirpation alone may be the definitive treatment [16, 23]. Our study showed that tumour size > 10 cm was a prognostic factor for both poor OS and CSS, which was consistent with a recent study [16].

### Survival for stage and surgery

Stage and surgery have been previously recognized as predictors of survival in patients with malignant bone tumours [24–26]. These factors were also independent predictors of OS and CSS in the study. MFN was highly metastatic tumour. Therefore, surgical removal of the primary tumour and distant lesions should be addressed and may be applicable to prolong survival in patients with MFN of the long bones and metastasis at diagnosis. Surgery remains the primary treatment strategy for MFN. Patients treated with this strategy had the best OS and CSS. Finally, there was not significant difference between upper limbs and lower limbs (data not shown).

### Strengths and limitations

This study is a population-based, with high-quality data, and the largest ever published on this category of rare tumours. However, this study has several limitations. These include the fact that there were no data on surgical type, node status, and RT dose.

## Conclusion

This is the largest population-based study to show demographics and analyse the prognosis of patients with MFN of the long bones. Independent predictors of OS included age, stage, tumour size and surgery. Age, stage, tumour size and surgery were also independent predictors of CSS. The results of this study may improve doctors’ understanding of the features and outcomes of MFN of the long bones. It may also be useful for patient health education and to provide a foundation for future research.

## Data Availability

The public can obtain the raw data from the author by emailing Yiting Huang.

## Abbreviations

CK: Cytokeratin
CSS: Cancer-specific survival
GCT: Giant cell tumour
LBFS: Fibrosarcoma of long bone
MFH: The malignant fibrous histiocytoma
MFN: Malignant fibrous neoplasms
mo: Months
NA: Not available
NED: No evidence of disease
NSAIDs: Non-steroidal anti-inflammatory drugs
OS: Overall survival
PVNS: Pigmented villonodular synovitis
ROM: Range of motion
SC: Synovial chondromatosis
SEER: The Surveillance, Epidemiology, and End Results
SMA: Smooth muscle actin
yr: Years

## References

[1] Belal A, Kandil A, Allam A, Khafaga Y, El-Husseiny G, El-Enbaby A, Memon M, Younge D, Moreau P, Gray A (2002). Malignant fibrous histiocytoma: a retrospective study of 109 cases. Am J Clin Oncol.

[2] Heffner DK, Gnepp DR (1992). Sinonasal fibrosarcomas, malignant schwannomas, and “triton” tumors. A clinicopathologic study of 67 cases. Cancer.

[3] Ozzello L, Stout AP, Murray MR (1963). Cultural characteristics of malignant histiocytomas and fibrous xanthomas. Cancer.

[4] Kauffman SL, Stout AP (1961). Histiocytic tumors (fibrous xanthoma and histiocytoma) in children. Cancer.

[5] Simons A, Schepens M, Jeuken J, Sprenger S, van de Zande G, Bjerkehagen B, Forus A, Weibolt V, Molenaar I, van den Berg E (2000). Frequent loss of 9p21 (p16(INK4A)) and other genomic imbalances in human malignant fibrous histiocytoma. Cancer Genet Cytogenet.

[6] Fu YS, Perzin KH (1976). Nonepithelial tumors of the nasal cavity, paranasal sinuses, and nasopharynx. A clinicopathologic study. VI. Fibrous tissue tumors (fibroma, fibromatosis, fibrosarcoma). Cancer.

[7] Ozkurt B, Basarir K, Yildiz YH, Kalem M, Saglik Y (2016). Primary malignant fibrous histiocytoma of long bones: long-term follow-up. Eklem Hastalik Cerrahisi.

[8] Niu Q, Lu Y, Xu S, Shi Q, Guo B, Guo Z, Huang T, Wu Y, Yu J (2018). Clinicopathological characteristics and survival outcomes of bladder neuroendocrine carcinomas: a population-based study. Cancer Manag Res.

[9] Ge LP, Liu XY, Xiao Y, Gou ZC, Zhao S, Jiang YZ, Di GH (2018). Clinicopathological characteristics and treatment outcomes of occult breast cancer: a SEER population-based study. Cancer Manag Res.

[10] Wu G, Wu J, Wang B, Zhu X, Shi X, Ding Y (2018). Importance of tumor size at diagnosis as a prognostic factor for hepatocellular carcinoma survival: a population-based study. Cancer Manag Res.

[11] Pan Y, Lu L, Chen J, Zhong Y, Dai Z (2018). Analysis of prognostic factors for survival in patients with primary spinal chordoma using the SEER registry from 1973 to 2014. J Orthop Surg Res.

[12] Ren S, Wang Z, Huang X, Sun L, Shao J, Ye Z (2018). Prognostic factors for postoperative survival among patients with rhabdomyosarcoma of the limbs. Cancer Manag Res.

[13] Patel TD, Carniol ET, Vazquez A, Baredes S, Liu JK, Eloy JA (2016). Sinonasal fibrosarcoma: analysis of the surveillance, epidemiology, and end results database. Int Forum Allergy Rhinol.

[14] Rubio GA, Alvarado A, Gerth DJ, Tashiro J, Thaller SR (2017). Incidence and outcomes of Dermatofibrosarcoma Protuberans in the US pediatric population. J Craniofac Surg.

[15] Tseng WH, Martinez SR, Do L, Tamurian RM, Borys D, Canter RJ (2011). Lack of survival benefit following adjuvant radiation in patients with retroperitoneal sarcoma: a SEER analysis. J Surg Res.

[16] Borucki RB, Neskey DM, Lentsch EJ (2018). Malignant fibrous histiocytoma: database review suggests a favorable prognosis in the head and neck. Laryngoscope.

[17] Tseng W, Martinez SR, Tamurian RM, Borys D, Canter RJ (2012). Histologic type predicts survival in patients with retroperitoneal soft tissue sarcoma. J Surg Res.

[18] Campschroer T, van der Kwast TH, Jonges GN, Lock MT (2014). Angiosarcoma of the prostate: a more frequent finding in the future owing to radiotherapy? A literature review with treatment implications based on a case report. Scand J Urol.

[19] Matushansky I, Dela CF, Insel BJ, Hershman DL, Neugut AI (2013). Chemotherapy use in elderly patients with soft tissue sarcoma: a population-based study. Cancer Investig.

[20] Doyle LA (2014). Sarcoma classification: an update based on the 2013 World Health Organization classification of tumors of soft tissue and bone. Cancer.

[21] Wang Z, Li S, Li Y, Lin N, Huang X, Liu M, Pan W, Yan X, Sun L, Li H (2018). Prognostic factors for survival among patients with primary bone sarcomas of small bones. Cancer Manag Res.

[22] Williams N, Morris CG, Kirwan JM, Dagan R, Mendenhall WM (2014). Radiotherapy for dermatofibrosarcoma protuberans. Am J Clin Oncol.

[23] Salerni G, Alonso C, Sanchez-Granel G, Gorosito M (2017). Dermoscopic findings in an early malignant fibrous histiocytoma on the face. Dermatol Pract Concept.

[24] Arshi A, Sharim J, Park DY, Park HY, Bernthal NM, Yazdanshenas H, Shamie AN (2017). Chondrosarcoma of the osseous spine: an analysis of epidemiology, patient outcomes, and prognostic factors using the SEER registry from 1973 to 2012. Spine (Phila Pa 1976).

[25] Arshi A, Sharim J, Park DY, Park HY, Yazdanshenas H, Bernthal NM, Shamie AN (2017). Prognostic determinants and treatment outcomes analysis of osteosarcoma and Ewing sarcoma of the spine. Spine J.

[26] Strotman PK, Reif TJ, Kliethermes SA, Sandhu JK, Nystrom LM (2017). Dedifferentiated chondrosarcoma: a survival analysis of 159 cases from the SEER database (2001-2011). J Surg Oncol.

